# Stomach microbiota, *Helicobacter pylori*, and group 2 innate lymphoid cells

**DOI:** 10.1038/s12276-020-00485-8

**Published:** 2020-09-10

**Authors:** Hiroshi Ohno, Naoko Satoh-Takayama

**Affiliations:** 1 Laboratory for Intestinal Ecosystem, RIKEN Center for Integrative Medical Sciences, 1-7-22 Suehiro-cho, Tsurumi-ku, Yokohama, Kanagawa 230-0045 Japan; 2 Intestinal Microbiota Project, Kanagawa Institute of Industrial Science and Technology, 3-2-1 Sakado, Takatsu-ku, Kawasaki, Kanagawa 213-0012 Japan; 3 Immunobiology Laboratory, Graduate School of Medical Life Science, Yokohama City University, 1-7-29 Suehiro-cho, Tsurumi-ku, Yokohama, Kanagawa 230-0045 Japan; 4 Laboratory for Immune Regulation, Graduate School of Medicine, Chiba University, 1-8-1 Inohana, Chuo-ku, Chiba, 260-8670 Japan

**Keywords:** Mucosal immunology, Innate lymphoid cells

## Abstract

The stomach has been thought to host few commensal bacteria because of the existence of barriers, such as gastric acid. However, recent culture-independent, sequencing-based microbial analysis has shown that the stomach also harbors a wide diversity of microbiota. Although the stomach immune system, especially innate lymphoid cells (ILCs), has not been well elucidated, recent studies have shown that group 2 ILCs (ILC2s) are the dominant subtype in the stomach of both humans and mice. Stomach ILC2s are unique in that their existence is dependent on stomach microbiota, in sharp contrast to the lack of an impact of commensal microbiota on ILC2s in other tissues. The microbiota dependency of stomach ILC2s is partly explained by their responsiveness to interleukin (IL)-7. Stomach ILC2s express significantly higher IL-7 receptor protein levels on their surface and proliferate more in response to IL-7 stimulation in vitro than small intestinal ILC2s. Consistently, the stomach expresses much higher IL-7 protein levels than the small intestine. IL-5 secreted from stomach ILC2s promotes immunoglobulin (Ig) A production by plasma B cells. In a murine model, stomach ILC2s are important in containing *Helicobacter pylori* infection, especially in the early phase of infection, by promoting IgA production.

## Gut microbiome: a surprise in the stomach

The stomach’s microbiota, and its effect on immune development, may be key factors in fending off infection with *Helicobacter pylori*, a bacterium found in the stomach that can lead to gastric upset and, if left untreated, ulcers or stomach cancer. Unlike the intestines, the stomach was thought to contain few bacteria owing to its high acidity, but according to new studies, the stomach harbors its own set of microbiota. Hiroshi Ohno and Naoko Satoh-Takayama at the RIKEN Center for Integrative Medical Sciences and Yokohama City University, Japan, review how gastric microbiota affects development of immune cells in the stomach. They report that the stomach’s unique microbiota influences maturation of particular immune cells, which may be crucial in fighting off *H. pylori* infection. These findings illuminate a new area in microbiome research.

## Introduction

The surfaces of an animal’s body, such as the skin and mucosa, provide a boundary between the body and the surrounding environment and are colonized by numerous commensal bacteria, called microbiota. In particular, the intestinal mucosa hosts the largest microbial community, of 40 trillion bacteria, in the body^1^. Gut microbiota contribute to gut maturation^2,3^, host nutrition, and pathogen resistance^4^. By contrast, it has been thought that there are fewer commensal bacteria in the stomach due to the characteristic feature of the gastric barrier, such as the highly acidic conditions of the lumen due to acid-secreting parietal cells and the presence of proteolytic enzymes (pepsins) from pepsinogen-secreting zymogenic chief cells^5,6^. The impact of gastric microbiota on host pathophysiology is not well understood compared to the microbiota of the intestine. Compared to the intestine, where evidence has accumulated that it has evolved the unique gut-associated immune system to deal with gut microbiota and orally ingested pathogens^7,8^, studies on the stomach immune system are less common. However, recent studies have shown that wide bacterial diversity can be found in the human stomach as well^5,9^^,^^10^^,^^11^.

Here, we will review the recently identified characteristics of stomach group 2 innate lymphoid cells (ILC2s), especially their relationship with gastric microbiota and *Helicobacter pylori* infection.

## Stomach and microbiota

Recent culture-independent analyses of microbial composition with 16 S rRNA sequencing have shown wide bacterial diversity in the human stomach^5,9–11^. In individuals who have neither of the two major gastric microbiota-perturbing events, *H. pylori* infection nor proton pump inhibitor (PPI) usage^5^, it has been shown that the microbiota in the stomach (and the duodenum) possesses a significantly lower number of bacteria than that of the lower gastrointestinal tract, which is probably correlated with the lower luminal pH in these organs^6^. The major phyla are *Firmicutes*, *Bacteroidetes*, *Actinobacteria*, and *Proteobacteria* throughout the human gastrointestinal tract^5,11^. In contrast to the lower intestinal tract (and feces), where *Firmicutes* are dominant, followed by *Bacteroidetes*^11,12^, *Firmicutes*, and *Proteobacteria* are dominant in the stomach and the duodenum^5,9,10^. At the genus level, *Streptococcus* and *Prevotella* are the two major genera detected in the stomach^9,10^, which are minor genera in human feces, considered to reflect their decreased presence in the lower gastrointestinal tract^12^.

## Gastric-microbiota-modulating factors: *H. pylori* and PPIs

As mentioned above, *H. pylori* infection perturbs the gastric microbiota^5^. *H. pylori* is a Gram-negative bacterium categorized as a pathobiont bacteria that colonizes the gastric mucosa of half or more of the human population in the world, ranging from 20% in developed countries to >90% in developing countries^5,13,14^. *H. pylori* colonizes the gastric mucosa using virulence properties, such as vacuolating cytotoxin, VacA and cytotoxin-associated gene A, cagA, and causes acute and chronic gastritis, eventually progressing to more severe disorders, including peptic ulcer disease and gastric cancer^15^.

*H. pylori* needs to overcome harsh acidic conditions in the stomach lumen to colonize and establish infection in the stomach^5,13^. It has been well documented that *H. pylori* infection occurs in early childhood, but rarely in adults^13^. *H. pylori* infection is basically acquired in childhood and persists throughout life without eradication therapy^16,17^. One of the possible explanations is that the ontogenetic development of the stomach in early childhood life is thought to be associated with the alleviation of gastric hostility, including achlorhydria, compared to adulthood^18^. Gastric hostility in adults sufficiently inhibits *H. pylori* infection. Therefore, *H. pylori* infection rarely occurs in adulthood, and when it does, it likely causes acute gastritis and is then eradicated^19,20^. *H. pylori* possesses urase and other enzymes that produce ammonia, which aid its infection by increasing the pH^13,19^.

Chronic *H. pylori* infection causes atrophic gastritis with a loss of parietal cells^5,13^. This leads to increased gastric pH and could modulate the gastric microbiota. The impact of *H. pylori* infection on the gastric microbiota has been studied. Although the majority of the gastric microbiota is occupied by *H. pylori*, its presence does not largely affect the composition of the gastric community^9^. *H. pylori* infection-associated gastric microbiota dysbiosis has been implicated in the pathogenesis of gastric diseases^21,22^, but the understanding of the complex interaction between the microbiota and *H. pylori* still needs to be further addressed in the development of gastric diseases.

PPIs are another gastric microbiota modifier^5^. PPIs suppress gastric acid secretion, are among the most commonly prescribed drugs and are widely used to treat gastric acid-related disorders, such as gastroesophageal reflux disease and peptic ulcers^23,24^. PPIs reduce bactericidal gastric acid secretion and lead to a significant change in the overall microbiota composition^11,23^. In the stomach, with PPI treatment, a greater diversity of bacterial species is observed, and the relative abundance of the phyla *Firmicutes* and *Fusobacteria* (whose abundance is very small without PPIs) is increased^11^.

## ILCs and gut microbiota

**Fig. 1:**
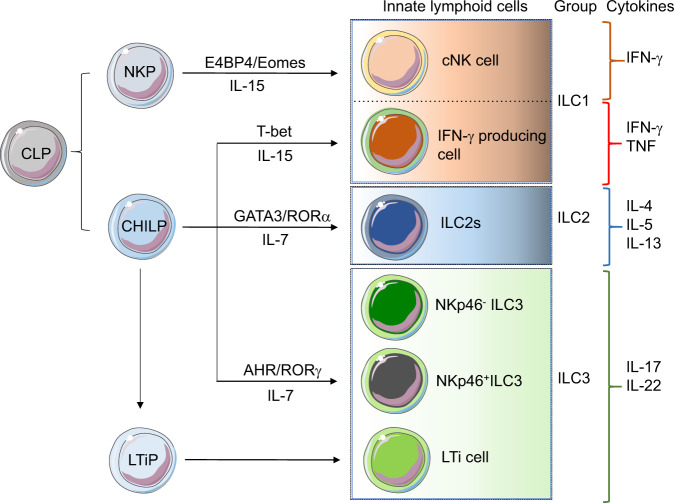
Characteristics of ILC subsets. Outline of ILC differentiation pathways with the respective transcription factors required for their differentiation and cytokines produced by ILC subsets. CLP common lymphoid progenitor, NKP NK cell progenitor, CHILP common helper-like innate lymphoid precursor, E4BP4 E4 promoter-binding protein 4 (also known as NFIL3), ROR RAR-related orphan receptor, AHR aryl hydrocarbon receptor, cNK conventional NK cell, LTiP lymphoid tissue inducer projenitors, LTi lymphoid tissue inducer.

ILCs are largely classified into three groups based on the characteristic expression of transcription factors and cytokine production (Fig. 1)^25,26^. ILC1s used to include all ILCs producing interferon-γ; however, it is now common to say that ILC1s are distinct from NK cells, whose differentiation is regulated by E4BP4 and Eomes^25,26^. ILC3s are known to produce interleukin (IL)-17 and IL-22 upon stimulation with IL-1β or IL-23; they are subdivided into (i) lymphoid tissue-inducer cells, which express CCR6 and possess lymphoid tissue-inducing ability; (ii) NKp46-ILC3s, which express NKp46 (also called natural cytotoxicity receptor 1); and (iii) CCR6^−^NKp46^−^ILC3s, which do not belong to (i) and (ii)^27^. ILC3s are basically distributed in tissues facing the external environment, especially the gastrointestinal tract, and play a role in defense mechanisms against pathogens^27^. Although the relationship between ILCs and gut microbiota has not been well understood until recently, recent advances in ILC biology, including single-cell analysis, have unveiled the details of the relationship^28^.

## ILC2s in physiology and pathology

ILC2s were independently identified in 2010 by three groups^29–31^. ILC2s produce type 2 cytokines, such as IL-5, IL-9, and IL-13, upon stimulation with IL-25, IL-33 or TSLP, and are involved in the eradication of parasite infection^29–31^. Recent studies have attracted attention on ILC2s by showing that these cells are involved in the pathogenesis of diseases, including fibrosis, asthma, and obesity^32–36^. In these diseases, the common scheme of pathogenesis is that an alarmin, IL-33, released upon epithelial cell damage by allergens, inflammation, or stress responses, triggers the activation of ILC2s. IL-33-activated ILC2s secrete IL-4, IL-5, and IL-9, which, in turn, induce eosinophils and activate mast cells, while IL-13 from ILC2s induces fibrosis^37–39^. In addition, it has been reported that ILC2s produce not only cytokines, but also opioid methionine-enkephalin peptides to regulate adipose function and metabolic homeostasis by acting directly on adipocytes^35^. In contrast to ILC3s, commensal microbiota do not seem to be involved in the induction or differentiation of ILC2s^40,41^. These observations were made with ILC2s in adipose tissues, the intestine, and the lung, which is in sharp contrast to the microbiota dependency observed with stomach ILC2s as described below.

## ILC2s and stomach microbiota

Studies on ILCs have mostly been performed in the intestine and lung, but information on the distribution and function of ILCs in the stomach is still lacking. We have recently examined ILC subsets in the murine stomach^42^. In the stomach, ILC2s are dominant, with a few ILC1s, and the stomach is almost devoid of ILC3s. This distribution is in sharp contrast to that observed in the intestine, where comparable numbers of the three ILC subsets exist. ILC2 dominance in the stomach has also been reported in humans^43^.

**Fig. 2:**
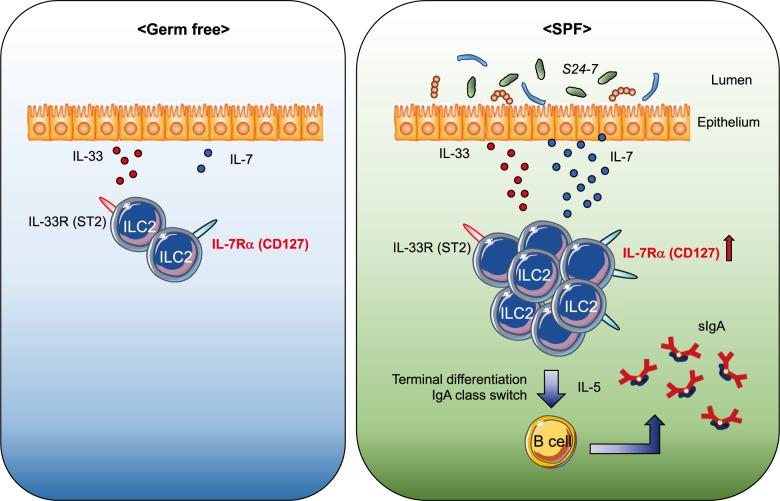
Stomach ILC2s. In germ-free (GF) mice, there are few ILC2s. Under GF conditions, IL-7 expression in stomach tissue is hardly detectable, and IL-7 receptor expression on stomach ILC2s is lower than that under SPF conditions. In contrast, the expression of the IL-33 receptor on stomach ILC2s is similar and that of IL-33 in the stomach tissue is slightly decreased in GF compared to SPF conditions. Under SPF conditions, the combination of IL-33 and IL-7 signaling leads to IL-5 secretion by ILC2s, which then promotes IgA production from plasma B cells.

More importantly, ILC2s existing in the stomach are dependent on stomach microbiota^42^, unlike ILC2s in the other tissues/organs. In germ-free (GF) mice, the number of stomach ILC2s was significantly decreased (Fig. 2). This observation is also in sharp contrast to the intestine and the lung, where the number of ILC2s is not affected by a lack of a microbiota, as seen in GF mice^40,41^.

Our findings indicate that stomach ILC2s possess unique characteristics compared to their counterparts in other tissues^42^. In fact, stomach ILC2s express significantly higher IL-7R levels on their surface and proliferate more in response to IL-7 stimulation than SI ILC2s in vitro. Consistently, the stomach expresses much higher IL-7 protein levels than the small intestine. Interestingly, the expression of IL-7 in stomach tissue is hardly detected under GF conditions. In addition, IL-7R expression on stomach ILC2s is also dependent on the existence of stomach microbiota, at least partly explaining the microbiota dependency of stomach ILC2s. In contrast, the expression of the IL-33 receptor on stomach ILC2s is comparable, and that of IL-33 in the stomach tissue is slightly decreased in GF compared to specific pathogen-free (SPF) conditions (Fig. 2)^42^.

Immunoglobulin (Ig) A coats the surface of commensal bacteria to prevent them from attaching to and invading the intestinal epithelium^44^. With the help of cytokines, B cells undergo class switch recombination to become different classes of Ig-producing B cells. In the case of IgA, IL-4 regulates class switching of B cells from IgM to IgA, and IL-5 further promotes the differentiation of IgA-expressing B cells to IgA-secreting plasma cells^45^. Although T helper (Th) 2 cells are the main source of IL-5, ILC2s have recently been established as another source of robust IL-5 secretion^29,30,31,46^. We have shown that microbiota-induced ILC2s are required as a major source of IL-5 for the induction of IgA^+^ plasma B cells, as well as efficient IgA secretion from these cells in the stomach (Fig. 2)^42^. The secreted IgA could modulate stomach microbiota composition by coating its subset.

Among stomach microbiota, members of the *S24-7* family within the order *Bacteroidales* are suggested to be candidate bacteria for ILC2 induction^42^. Our preliminary results indicate that there seems to be differential ILC2-inducing ability among several *S24-7* clones, and further studies should clarify the molecular mechanisms underlying bacterial induction of stomach ILC2s.

PPIs suppress gastric acid secretion and are among the most commonly prescribed drugs, widely used to treat gastric acid-related disorders, such as gastroesophageal reflux disease and peptic ulcer and, sometimes, *H. pylori* infection^23,24^. PPIs reduce bactericidal gastric acid secretion and lead to a significant change in the overall microbiota composition^11,23^. In the stomach, with PPI treatment, a greater diversity of bacterial species is observed, and the relative abundance of the phyla *Firmicutes* and *Fusobacteria* (whose abundance is very small without PPIs) is increased^11^. This change in gastric microflora composition might affect the number of ILC2-inducible bacteria and, thus, the number of ILC2s in the stomach, which, in turn, can alter the composition of the gastric microbiota.

## Stomach ILC2s and *H. pylori* infection

**Fig. 3:**
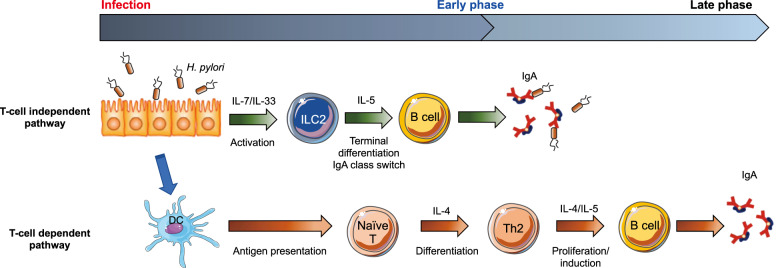
The role of stomach ILC2s in *H. pylori* infection. In the early phase of *H. pylori* infection, the induction of IL-7 and IL-33 in the stomach tissue increases and activates ILC2s to secrete IL-5, which promotes plasma B cells differentiation and IgA production. At this stage, there is no apparent increase in CD4^+^T cells (T-cell-independent pathway). In the later phase of the infection, IL-5 derived from Th2-type CD4^+^ T cells also promotes IgA production (T-cell dependent pathway).

*H. pylori* infection, especially in the acute phase, is accompanied by a mucosal IgA response, although the T-cell response is predominantly Th1 type^16^^,^^47,48^. In a murine *H. pylori* infection model, we recently identified that stomach ILC2s are the source of IL-5 for IgA production in the early phase of *H. pylori* infection (Fig. 3)^42^. The expansion of IL-5-receptor-expressing plasmablasts is initiated by ILC2 activation, which is indispensable to restrain *H. pylori* infection. This is also supported by the fact that a significant reduction in plasma B cells and plasmablasts, as well as a reduction in *H. pylori* coated with IgA, was observed in mice with ILC2-specific deficiency. IgA-mediated *H. pylori* elimination has a strong correlation with the restraint of gastric inflammation, which is prominently diminished in mice lacking ILC2s^42^. It has also been shown that the Th2 cell-dependent IgA response is induced for mucosal protection in the late phase of infection (Fig. 3)^42^.

As mentioned above, *H. pylori* infection is often acquired in childhood and persists throughout life without eradication therapy^44,46^, while *H. pylori* infection rarely occurs in adulthood, and when it does, it causes acute gastritis and is then eradicated^47,48^. Since early childhood is a particularly vulnerable period as the immune system is still developing, one bold hypothesis is that not enough ILC2 development occurs to eradicate *H. pylori* infection in childhood, although there is no evidence to support this notion at this time; further studies are awaited to clarify whether this hypothesis is true.

## Conclusion

It is now clear that the stomach is not a totally sterile and immunologically silent organ, but instead harbors a wide diversity of commensal microbiota with unique composition and a unique immune system within the gastrointestinal tract. In particular, it possesses ILC2s with characteristics that are distinct from their counterparts in other organs/tissues. The stomach ILC2s are dependent on the presence of stomach microbiota, and they can modulate the composition of the microbiota by promoting IgA production. Stomach ILC2s are also important in *H. pylori* infection. *H. pylori* can evade or disturb innate and adaptive immune responses via multiple mechanisms to establish chronic infection, leading to clinical gastroduodenal disorders, including chronic gastritis, peptic ulcers, and two malignancies—gastric adenocarcinoma and mucosa-associated lymphoid tissue lymphoma. Therefore, it is important to understand the stomach immune system, including ILC2s, in more detail to develop novel treatment strategies to contain its infection.
